# Excess body weight, weight gain and obesity-related cancer risk in women in Norway: the Norwegian Women and Cancer study

**DOI:** 10.1038/s41416-018-0240-5

**Published:** 2018-09-11

**Authors:** Marisa da Silva, Elisabete Weiderpass, Idlir Licaj, Lauren Lissner, Charlotta Rylander

**Affiliations:** 1 Department of Community Medicine, UiT The Arctic University of Norway, Tromsø, Norway; 2 Department of Research, Cancer Registry of Norway, Institute of Population-based Cancer Research, Oslo, Norway; 3 Department of Medical Epidemiology and Biostatistics, Karolinska Institutet, Stockholm, Sweden; 4 Genetic Epidemiology Group, Folkhälsan Research Center and Faculty of Medicine, University of Helsinki, Helsinki, Finland; 5 Clinical Research Department, Centre François Baclesse, Caen, France; 6 Section for Epidemiology and Social Medicine, Institute of Medicine, Sahlgrenska Academy, University of Gothenburg, Gothenburg, Sweden

**Keywords:** Risk factors, Cancer epidemiology

## Abstract

**Background:**

Excess body weight and weight gain have been reported to independently increase the risk of several cancers. There are few published studies in nationally representative populations of women on specific, ‘obesity-related’ cancers in relation to prior weight change and relevant confounders.

**Methods:**

Based on self-reported anthropometry, we prospectively assessed body mass index (BMI), weight change over 6 years and subsequent obesity-related cancer risk in the Norwegian Women and Cancer study. We used Cox proportional hazard models to calculate hazard ratios and restricted cubic splines to model potential non-linear dose–response relationships.

**Results:**

Excess body weight increased the risk of overall obesity-related cancer, postmenopausal breast, colorectal, colon, endometrial and kidney cancer, with endometrial cancer showing a threefold elevated risk. High weight gain ( ≥ 10 kg) increased the risk of overall obesity-related cancer, postmenopausal breast, endometrial and pancreatic cancer. The association between high weight gain and pancreatic cancer was strong, with 91% increased risk.

**Conclusions:**

Maintaining stable weight in middle adulthood, irrespective of BMI category at baseline, and avoiding excess body weight are both important in the prevention of several obesity-related cancers in women. Our finding of increased risk of pancreatic cancer in women with moderate and high weight gain is novel.

## Background

The prevalence of overweight and obesity has been increasing continuously worldwide over the past four decades.^1^ Although body weight is a modifiable factor, the attempts to halt the obesity epidemic has failed. The global burden of cancer has increased alongside the obesity prevalence, with 13 cancer types defined as obesity-related.^2,3^ The cancers with sufficient evidence of a positive association with overweight or obesity (also referred to as excess body weight) are cancer of the breast (postmenopausal), colon–rectum, endometrium, ovary, pancreas, kidney, gallbladder, gastric cardia, liver, oesophagus (adenocarcinoma), meningioma, thyroid and multiple myeloma. Weight gain is also associated with several obesity-related cancers independent of body composition.^4^ However, nationally representative studies on weight gain and the risk of less-commonly diagnosed obesity-related cancers such as pancreatic and kidney cancer in women are rare. In fact, in the latest report from The World Cancer Research Fund’s Continuous Update Project, the expert panel concludes that postmenopausal breast cancer is the only cancer for which there is strong evidence of an association with weight gain.^5^ Thus, there is an evident research gap on weight gain and specific obesity-related cancers.

In accordance with global trends, there are indications of increased obesity prevalence in Norway. The latest regional health examination from Nord-Trøndelag (HUNT), carried out in 2006–2008, reported a prevalence of obesity of 23.1% in women. This represented a 10%-point increase from the previous HUNT report covering the period 1984–1986.^6^ In addition, Statistics Norway conduct a survey on living conditions every 3 years in a representative sample of inhabitants in Norway aged 16 years or older.^7^ Since 1998, the self-reported prevalence of obesity has increased in both women and men and reached 11% in women in 2015. Surely, there are differences in obesity prevalence according to age, region, rural/urban settlements and reporting method (self-report or examination), however, there is little doubt that increasing body weight is a public health concern also in Norway. Moreover, three of the five most commonly diagnosed cancers among women in Norway are obesity-related (breast, colon and endometrial cancer) and the overall cancer incidence rate has increased.^8^

In this study, we aimed to quantify separate risk estimates for body mass index (BMI) and short-term weight change in a nationally representative female cohort, for a large number of obesity-related cancers, including pancreatic and kidney cancer.

## Materials and methods

### Study design, participants and subsamples

The Norwegian Women and Cancer (NOWAC) study is a nationally representative, population-based cohort study that was initiated in 1991, with the aim of investigating the aetiology of cancer among women in Norway. Women aged 30–70 years were randomly sampled from the Norwegian Central Population Register, which includes all Norwegian inhabitants, and invited to participate in the study during three separate waves of recruitment: 1991–1992, 1996–1997 and 2003–2005. Those who agreed to participate completed an enrolment questionnaire (Q1) and were invited to complete a follow-up questionnaire (Q2) 5–8 years after Q1. The response rate in the NOWAC study varied between 48 and 57% at enrolment, and was 81% at follow-up. The unique personal identity number assigned to every resident of Norway allowed for linkages to national registers for complete follow-up.^9^ The external validity in NOWAC is considered high as the performed validation study showed that the distribution of exposures was independent of the response rate and the observed cumulative incidence of cancer vs expected national figures from the Cancer Registry of Norway showed no substantial differences.^10^ Details on the design, materials and procedures of the NOWAC study have been described elsewhere.^11^

**Fig. 1:**
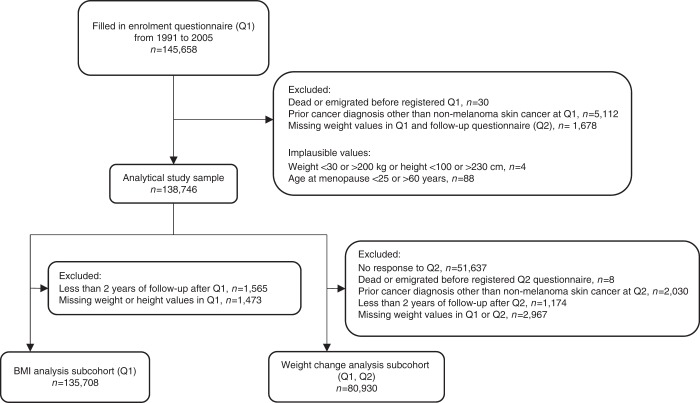
Flowchart of study participants

In the present study, 145,658 women who returned Q1 between 1991 and 2005 were considered eligible for inclusion (Fig. 1). We excluded women who had emigrated or died before Q1 was registered in the study database (*n* = 30), women who were diagnosed with cancer (other than non-melanoma skin cancer) prior to Q1 (*n* = 5112), and women with missing weight in both Q1 and Q2 (*n* = 1678). Women who reported implausible weight values ( < 30 or > 200 kg), height values ( < 100 or > 230 cm) (*n* = 4) or age at menopause ( < 25 or > 60 years) (*n* = 88) in either questionnaire were also excluded. Thus, our final analytical study sample consisted of 138,746 women: 40% enroled in 1991–1992, 31% enroled in 1996–1997 and 29% enroled in 2003–2005. BMI and weight change analyses were carried out in subsamples of the final analytical study sample. In the BMI analysis, we excluded women with < 2 years of follow-up after Q1 to reduce the possible influence of reverse causality from the effects of pre-clinical cancer on weight (*n* = 1 565), and women with missing weight or height in Q1 (*n* = 1473). In the weight change analysis, we excluded women who did not return Q2 (*n* = 51 637). Women who returned Q2 were younger, had lower body weight and were less likely to use hormone therapy (HT) compared with women who completed only Q1. Furthermore, we excluded women who emigrated or died before Q2 was registered in the study database (*n* = 8). Women who had been diagnosed with cancer (other than non-melanoma skin cancer) prior to Q2 (*n* = 2030), had < 2 years of follow-up after Q2 (*n* = 1174), or had missing information on weight in Q1 or Q2 were also excluded (*n* = 2967).

In site-specific analyses, we excluded premenopausal women from the postmenopausal breast cancer analysis (BMI analysis, *n* = 76,377; weight change analysis *n* = 34,222), women who reported hysterectomy from the endometrial cancer analysis (BMI analysis, *n* = 7394; weight change analysis, *n* = 5035) and women who reported bilateral oophorectomy from the ovarian cancer analysis (BMI analysis *n* = 2341, weight change analysis *n* = 1907).

### Follow-up and identification of cancer cases

Follow-up began at Q1 for the BMI analysis and at Q2 for the weight change analysis. Women were followed-up until cancer diagnosis, death, emigration or the end of follow-up (31 December 2014), whichever occurred first. Incidence of cancer, death and emigration were identified through linkage to the Norwegian Cancer Registry, the Cause of Death Registry and the Norwegian Central Population Register, respectively. The outcome of interest was first primary invasive cancer, for which evidence of a positive association with excess body weight is considered sufficient,^2^ hereafter, referred to as ‘obesity-related cancer’. These cancers were assessed as one combined outcome (overall obesity-related cancer) and as site-specific outcomes, and were classified according to the International Classification of Diseases, 10th Revision. They included cancer of the breast (postmenopausal) (C50), colon–rectum (C18–20), endometrium (C54), ovary (C56), pancreas (C25), kidney (C64), gallbladder (C23–24), gastric cardia (C16), liver (C22), oesophagus (adenocarcinoma) (C15), meningioma (C70–72), thyroid (C73) and multiple myeloma (C90). In the overall obesity-related cancer analysis, women were considered to have postmenopausal breast cancer if they reported being postmenopausal in Q1, or if they gave an age at menopause that was earlier than their age at breast cancer diagnosis. Women with unknown menopausal status or missing information on age at menopause were considered to have postmenopausal breast cancer if they had reached 53 years of age at or before the time of breast cancer diagnosis. This age cutoff has been used previously to classify women as postmenopausal in the NOWAC study^12^ and represents ~ 80% of the women in our study population who reached natural menopause. We did not perform site-specific analyses for cancer of the gallbladder, gastric cardia, liver, oesophagus, meningioma, thyroid or multiple myeloma, owing to the small number of incident cases for each of these sites.

### Assessment of BMI, weight change and covariates

BMI was calculated as self-reported weight in kg divided by the square of self-reported height in metres and categorised according to the World Health Organisation definition:^13^ underweight (BMI < 18.5 kg/m^2^), normal weight (BMI 18.5 ≤ 25 kg/m^2^), overweight (BMI 25 ≤ 30 kg/m^2^), or obesity (BMI ≥ 30 kg/m^2^). We used self-reported weight from Q1 and Q2 to calculate weight change, which was categorised into five groups: weight loss ( ≤ 2 kg), stable weight (−2– < 2 kg), low weight gain (2– < 5 kg), moderate weight gain (5– < 10 kg) or high weight gain ( ≥ 10 kg).

Information on covariates was extracted from Q1 for the BMI analysis, and Q1 or Q2 for the weight change analysis. An a priori selection of covariates was done, based on findings from previous studies on BMI or weight change and obesity-related cancer, as well as previous reports from the NOWAC study. Thus, the covariates education ( < 10 years/10–12 years/ > 12 years), physical activity level (low/moderate/high), smoking status (never/former/current) and alcohol intake ( ≤ median/ > median g/day) were included in all analyses. In addition, we assessed smoking transition (cessation/restart/no change) and physical activity change (increase/decrease/no change) in all weight change analyses. The outcome-specific covariates that were common for postmenopausal breast, ovarian and endometrial cancer were age at menarche ( ≤ median/ > median age), parity/age at first full-term pregnancy (nullipara/unipara < 29 years/unipara ≥ 30/multipara < 29/multipara ≥ 30), oral contraceptive (OC) use (never/ever) and HT use (never/former/current). For postmenopausal breast cancer, maternal history of breast cancer (yes/no) was also included in the model, and for endometrial and ovarian cancer, menopausal status was also included in the model. Diabetes (yes/no) was evaluated as a potential confounder for endometrial, colorectal, pancreatic and kidney cancer; for colorectal cancer (as well as for colon and rectal cancer analysed separately) we assessed consumption of red and processed meat, fruits, vegetables, fibre and calcium categorised into tertiles (low/medium/high).

### Statistical analysis

Population characteristics by BMI status and weight change category were assessed using *χ* ^2^ tests for categorical variables and one-way analysis of variance or Kruskal–Wallis test for continuous variables. We used Cox proportional hazard regression models with age as the underlying time metric^14^ to estimate hazard ratios and 95% confidence intervals (CI) for the associations of BMI and weight change with obesity-related cancer risk. The reference groups were ‘normal weight’ and ‘stable weight’. To account for the calendar and birth cohort effect, we constructed a variable based on wave of enrolment and birth year (categorised into four groups) that was included in the Cox regression models, and allowed the baseline hazard function to vary between the groups but with equal coefficients across groups. The Cox models were built according to the ‘purposeful selection’ approach.^15^ In brief, we performed univariable Cox models for each covariate and included those that were significant at a 20% level in a multivariable model (the full model). Thereafter, we used Wald statistics to exclude covariates that were no longer significant in the full model, or did not change the coefficients of the exposure variable > 20%. Log-likelihood ratio tests were performed to compare goodness of fit between the reduced model and the full model. Covariates that remained in the reduced final models are presented in the footnotes of Tables 2 and 3. Participants with missing information on included covariates were excluded from the analyses. Tests based on Schoenfeld residuals showed no evidence of violation of the proportional hazard assumptions.^16^ We fitted two models per outcome; Model 1 controlled only for age (by time in the Cox regression) and Model 2 (main model) with adjustments by purposeful selection of covariates for each outcome separately. We tested for plausible interactions with log-likelihood ratio test, comparing reduced models with and without the interaction term. In all weight change analyses, we tested for interaction between BMI status and weight change category. In site-specific analyses where HT use or menopausal status was included as a covariate, we tested for interactions between these and each exposure. In order to model potential non-linear dose–response relationships, we fitted restricted cubic spline transformations (four knots) of the exposure variables.^17^ We evaluated non-linearity by testing the null hypothesis of equal spline coefficients. The knots were placed at equally spaced percentiles as recommended by Harrell (2001).^18^ All statistical analyses were performed using STATA version 15.1 (Stata Corp., College Station, TX, USA).

## Results

In total, 135,708 women were included in the BMI analysis and 80,930 women who also responded to Q2 were included in the weight change analysis (Fig. 1). In the BMI analysis, average follow-up time was 16.9 (standard deviation (SD) = 5.8) years, during which 9328 obesity-related cancers were diagnosed, with a mean age at diagnosis of 61.9 (SD = 7.9) years. In the weight change analysis, average follow-up time was 13.1 (SD = 4.2) years, during which 4831 obesity-related cancers were diagnosed, with a mean age at diagnosis of 63.0 (SD = 7.7) years. The average response time between Q1 and Q2 was 6.3 years (SD = 0.9) and did not differ substantially across weight change categories.

### Population characteristics

In the BMI analysis, the population mean (SD) age, weight and BMI were 48.2 (8.6) years, 66.7 (11.4) kg and 24.1 (3.9) kg/m^2^, respectively. The majority of women were of normal weight (64.6%), followed by overweight (25.5%), obesity (7.7%) and underweight (2.2%) (Table 1). Compared with the other BMI categories, women with obesity were older, and had lower education, physical activity level and alcohol intake. They were more likely to be never or former smokers, report lower age at menarche, younger at first full-term pregnancy, have three or more children, less likely to use OC and more likely to report former use of HT.

**Table 1:** Population characteristics by body mass index (BMI) category at enrolment

	BMI category (kg/m^2^)				
	N^a^	Underweight	Normal weight	Overweight	Obesity
Number of women, *n* (%)	135,708	3022 (2.2)	87,595 (64.6)	34,656 (25.5)	10,435 (7.7)
Obesity-related cancer, *n*	9328	173	5689	2603	863
*Characteristics at enrolment* ^*b*^					
Age (y), mean (SD)	135,708	44.1 (8.4)	46.9 (8.4)	50.8 (8.4)	51.5 (8)
Weight (kg), mean (SD)	135,708	49.3 (3.9)	61.4 (6.1)	74.2 (6.3)	91.0 (11.7)
Height (cm), mean (SD)	135,708	166.6 (5.6)	166.5 (5.6)	165.9 (5.7)	165.3 (5.8)
Education (y) %	128,948				
< 10		24.0	21.7	29.8	34.3
10–12		22.1	23.5	24.6	24.4
> 12		53.9	54.9	45.6	41.3
Physical activity level %	123,531				
Low		25.7	21.2	30.7	45.7
Moderate		37.5	42.2	42.6	37.0
High		36.8	36.7	26.7	17.4
Smoking status %	135,231				
Never smoker		27.2	34.4	37.8	40.0
Former smoker		21.3	31.8	35.7	36.2
Current smoker		51.6	33.8	26.5	23.8
Alcohol intake (g/day), median	128,046	1.6	1.9	1.5	0.9
Age at menarche (y), mean (SD)	133,625	13.7 (1.4)	13.4 (1.4)	13.2 (1.4)	12.9 (1.4)
Age at first full-term pregnancy (y), mean (SD)	123,592	24.7 (4.7)	24.1 (4.4)	23.6 (4.3)	23.4 (4.4)
Parity %	135,708				
Nulliparous		13.0	9.5	8.1	11.1
1–2 children		56.8	55.7	50.1	46.3
≥ 3 children		30.2	34.9	41.9	42.6
Oral contraceptive use %	131,415				
Never		38.2	40.6	49.8	54.4
Ever		61.8	59.4	50.2	45.6
Menopausal status %	135,708				
Premenopausal		64.0	55.3	37.1	31.6
Perimenopausal		4.2	4.8	5.6	6.6
Postmenopausal		25.9	32.9	50.1	54.7
Unknown		5.9	7.0	7.2	7.2
Age at menopause (y), mean (SD)	45,160	46.7 (5.9)	48.3 (4.8)	48.8 (4.7)	48.5 (5.2)
Hormone therapy use %	126,669				
Never		85.7	79.6	72.7	73.7
Former		5.6	8.2	12.9	14.4
Current		8.7	12.2	14.4	11.9

The Norwegian Women and Cancer study 1991–2005 (*n* = 135, 708)

^a^ *N* is the total amount of responses for the specific variable

^b^Overall differences between weight change categories were significant for all variables (*p* < 0.001)

*y* years, *SD* standard deviation

In the weight change analysis, the population mean (SD) age, weight and BMI in Q2 was 52.4 (8.5) years, 68.6 (11.5) kg and 24.8 (3.9) kg/m^2^, respectively. During the 6.3 years between Q1 and Q2, 9.7% of women lost weight, 29.3% had stable weight, 27.6% had low weight gain, 24.1% had moderate weight gain and 9.3% had high weight gain (Supplementary Information, Table S1). Population characteristics differed across these weight change categories. Women with high weight gain were younger and reported lower physical activity at Q1 compared with women with stable weight. Moreover, between Q1 and Q2, women with high weight gain were more likely to have stopped smoking, decreased their physical activity level and transitioned to menopause.

### BMI and obesity-related cancer risk

Compared with normal-weight women, women with overweight or obesity had an increased obesity-related cancer risk, with HRs of 1.09 (95% CI: 1.03–1.14) and 1.24 (95% CI: 1.14–1.34) (Table 2). In site-specific analyses, endometrial cancer displayed a significant association with obesity, with an almost threefold increased risk (HR = 2.78, 95% CI: 2.30–3.35), as well as a significant association with overweight (HR = 1.45, 95% CI: 1.24–1.68). Furthermore, excess body weight increased the risk of postmenopausal breast cancer (overweight HR = 1.13, 95% CI: 1.00–1.27) and the association with obesity was of borderline significance (HR = 1.20, 95% CI: 1.00–1.44; *p* = 0.05). In addition, excess body weight was significantly associated with colorectal (overweight HR = 1.12, 95% CI: 1.01–1.25), colon (overweight HR = 1.21, 95% CI: 1.07–1.37) and kidney cancer (obesity HR = 1.95, 95% CI: 1.26–3.02). An increment of five BMI units was significantly associated with increased risk of overall obesity-related cancer, postmenopausal breast cancer, endometrial and kidney cancer. There was no significant association between excess body weight and increased risk of rectal, ovarian and pancreatic cancer.

**Table 2:** Hazard ratio (HR) with 95% confidence interval (CI) for obesity-related cancer risk by body mass index (BMI) category at enrolment

	Model 1 age-adjusted				Model 2 multivariable			
	*N*	Cancer cases	HR	95% CI	*N*	Cancer cases	HR	95% CI
*Overall obesity-related cancer* ^*a*^								
Underweight	3022	173	0.95	0.82–1.10	2626	149	0.92	0.78–1.08
Normal weight	87,595	5689	1.00	Reference	77,064	4961	1.00	Reference
Overweight	34,656	2603	1.10	1.05–1.15	29,517	2154	1.09	1.03–1.14
Obesity	10,435	863	1.26	1.17–1.35	8706	699	1.24	1.14–1.34
5 BMI unit increment	135,708	9328	1.11	1.08–1.14	117,913	7 963	1.10	1.07–1.13
*Postmenopausal breast cancer* ^*b*^								
Underweight	899	27	0.96	0.66–1.41	650	19	0.98	0.62–1.55
Normal weight	32,831	1047	1.00	Reference	24,224	730	1.00	Reference
Overweight	19,270	638	1.04	0.95–1.15	14,079	448	1.13	1.00–1.27
Obesity	6331	206	1.07	0.92–1.24	4540	139	1.20	1.00–1.44
5 BMI unit increment	59,331	1918	1.03	0.97–1.08	43,493	1 336	1.07	1.00–1.15
*Colorectal cancer* ^*c*^								
Underweight	3022	39	1.11	0.80–1.52	2902	38	1.10	0.80–1.52
Normal weight	87,595	1 146	1.00	Reference	83,411	1083	1.00	Reference
Overweight	34,656	585	1.12	1.02–1.24	32,511	544	1.12	1.01–1.25
Obesity	10,435	157	1.05	0.88–1.24	9744	140	1.01	0.84–1.20
5 BMI unit increment	135,708	1 927	1.05	0.99–1.11	128,568	1 805	1.04	0.98–1.11
*Colon cancer* ^*d*^								
Underweight	3022	26	1.14	0.77–1.69	3017	26	1.13	0.76–1.67
Normal weight	87,595	746	1.00	Reference	87,355	743	1.00	Reference
Overweight	34,656	414	1.20	1.06–1.36	34,481	411	1.21	1.07–1.37
Obesity	10,435	104	1.05	0.85–1.29	10,378	103	1.06	0.86–1.30
5 BMI unit increment	135,708	1 290	1.06	0.99–1.14	135,231	1 283	1.07	0.99–1.14
*Rectal cancer* ^*e*^								
Underweight	3022	13	1.05	0.60–1.82	2805	11	0.99	0.54–1.81
Normal weight	87,595	400	1.00	Reference	79,948	354	1.00	Reference
Overweight	34,656	171	0.98	0.82–1.18	30,665	153	1.02	0.84–1.24
Obesity	10,435	53	1.05	0.78–1.40	9189	44	1.03	0.75–1.42
5 BMI unit increment	135,708	637	1.03	0.93–1.14	122,607	562	1.04	0.93–1.16
*Endometrial cancer* ^*f*^								
Underweight	2914	11	0.62	0.34–1.13	2594	10	0.63	0.34–1.18
Normal weight	83,620	539	1.00	Reference	74,239	489	1.00	Reference
Overweight	32,163	321	1.50	1.30–1.72	27,991	277	1.45	1.24–1.68
Obesity	9617	186	3.02	2.55–3.58	8326	156	2.78	2.30–3.35
5 BMI unit increment	128,314	1057	1.53	1.45–1.62	113150	932	1.51	1.42–1.60
*Ovarian cancer* ^*g*^								
Underweight	2991	11	0.75	0.41–1.36	2851	10	0.71	0.38–1.33
Normal weight	86,442	429	1.00	Reference	81,300	404	1.00	Reference
Overweight	33,816	149	0.91	0.75–1.10	31,608	142	0.92	0.76–1.12
Obesity	10,118	53	1.13	0.85–1.51	9425	49	1.09	0.81–1.48
5 BMI unit increment	133,367	642	1.01	0.91–1.12	125,148	605	1.00	0.90–1.12
*Pancreatic cancer* ^*c*^								
Underweight	3059	5	0.75	0.31–1.83	2902	4	0.55	0.20–1.48
Normal weight	88,480	213	1.00	Reference	83,411	202	1.00	Reference
Overweight	35,092	104	1.11	0.87–1.41	32,511	97	1.18	0.92–1.51
Obesity	10,574	28	1.05	0.70–1.56	9744	29	1.19	0.79–1.79
5 BMI unit increment	135,708	350	1.02	0.89–1.17	128,568	324	1.11	0.96–1.27
*Kidney cancer* ^*h*^								
Underweight	3059	2	0.40	0.10–1.60	2295	2	0.50	0.12–2.04
Normal weight	88,480	158	1.00	Reference	68,745	120	1.00	Reference
Overweight	35,092	94	1.41	1.08–1.82	26,124	62	1.32	0.96–1.81
Obesity	10,574	38	1.97	1.38–2.83	7502	27	1.95	1.26–3.02
5 BMI unit increment	135,708	292	1.34	1.18–1.51	104,666	211	1.33	1.15–1.54

^a^Model 2 for overall obesity-related cancer was adjusted for age, education, physical activity and smoking status

^b^Only in women who were postmenopausal at enrolment, model 2 for postmenopausal breast cancer was adjusted for age, education, alcohol intake, parity/age at first full-term pregnancy, oral contraceptive use, hormone therapy use and history of breast cancer in the mother

^c^Model 2 for colorectal and pancreatic cancer was adjusted for age, education and smoking status

^d^Model 2 for colon cancer was adjusted for age and smoking status

^e^Model 2 for rectal cancer was adjusted for age, education and alcohol intake

^f^Model 2 for endometrial cancer was adjusted for age, education, age at menarche, parity/age at first full-term pregnancy, oral contraceptive use and menopausal status

^g^Model 2 for ovarian cancer was adjusted for age, parity/age at first full-term pregnancy and oral contraceptive use

^h^Model 2 for kidney cancer was adjusted for age, smoking status and diabetes

The Norwegian Women and Cancer study, 1991–2014 (*n* = 135,708)

**Fig. 2:**
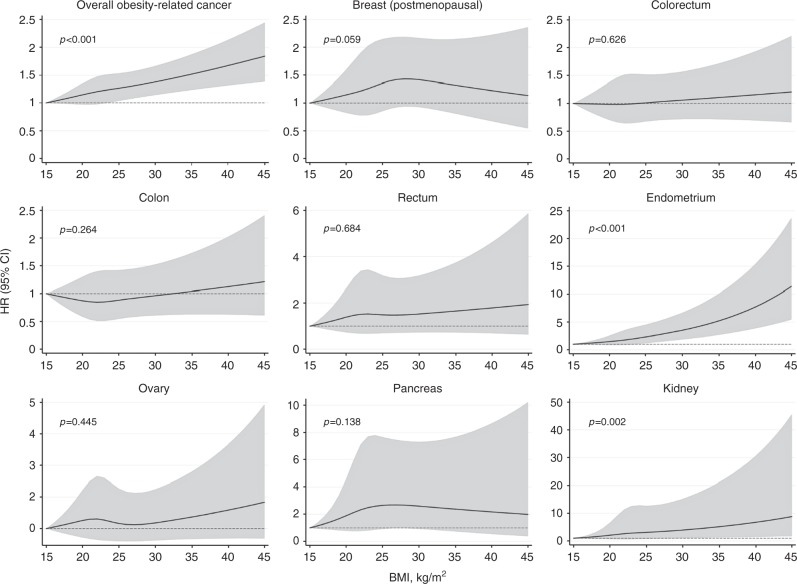
Non-linear effects of BMI at enrolment and risk of specific and overall obesity-related cancers, with 95% CI. Restricted cubic splines were fitted with knots at BMI 19, 22, 25 and 31. *P* values are for non-linearity

Further, a clear dose–response relationship with increasing BMI was found for overall obesity-related cancer, endometrial and kidney cancer (Fig. 2). These dose–response relationships were statistically significant at different BMI; kidney cancer was statistically significant only after BMI 30, whereas overall obesity-related cancer and endometrial cancer were statistically significant at BMI 24 (Supplementary Information, Table S3–5). We found no statistically significant interactions between HT use and BMI; however, menopausal status modified the effect of BMI in relation to endometrial cancer risk with a statistically significant interaction between perimenopausal status and obesity. We performed stratified analysis by menopausal status (Supplementary Information, Table S2) but the subgroup analysis result should be interpreted with caution due to the low number of cases (58) in the perimenopausal status group.

### Weight change and obesity-related cancer risk

Weight gain was significantly associated with increased obesity-related cancer risk, with associations observed among women with low weight gain (HR = 1.14, 95% CI: 1.05–1.23), moderate weight gain (HR = 1.14, 95% CI: 1.05–1.25) and high weight gain (HR = 1.16, 95% CI: 1.04–1.31), versus stable weight (Table 3). High weight gain was further significantly associated with nearly a twofold increased risk in pancreatic cancer (HR = 1.91, 95% CI: 1.11–3.30), also moderate weight gain increased the risk of pancreatic cancer (HR = 1.60, 95% CI: 1.03–2.47). Furthermore, weight gain increased the risk of postmenopausal breast cancer (moderate weight gain HR = 1.20, 95% CI: 1.01–1.43; high weight gain HR = 1.36, 95% CI: 1.08–1.71), as well as colorectal (moderate weight gain HR = 1.24, 95% CI: 1.05–1.48), rectal (low weight gain HR = 1.37, 95% CI: 1.00–1.86; moderate weight gain HR = 1.38, 95% CI: 1.00–1.91; *p* = 0.05) and endometrial cancer (moderate weight gain HR = 1.27, 95% CI: 1.01–1.61; high weight gain HR = 1.40, 95% CI: 1.04–1.88). Weight loss was significantly associated with an increased risk of colorectal cancer (HR = 1.25, CI: 1.01–1.55) and displayed positive associations with all obesity-related cancers under study, although they did not reach statistical significance. A 5 kg increase in weight was significantly associated with increased risk of overall obesity-related cancer, postmenopausal breast cancer and endometrial cancer. We found no significant association between weight change and the risk of colon, ovarian and kidney cancer.

**Table 3:** Hazard ratio (HR) with 95% confidence interval (CI) for obesity-related cancer risk by weight change category between the enrolment (Q1) and follow-up questionnaire (Q2)

	Model 1 age-adjusted				Model 2 Multivariable			
	N	Cancer cases	HR	95% CI	*N*	Cancer cases	HR	95% CI
*Overall obesity-related cancer* ^*a*^								
Weight loss (< −2kg)	7876	478	1.15	1.04–1.28	6886	406	1.09	0.97–1.22
Stable weight (−2– < 2 kg)	23,711	1315	1.00	Reference	20,950	1 142	1.00	Reference
Low weight gain (2– < 5 kg)	22,362	1356	1.10	1.02–1.19	19,844	1 209	1.14	1.05–1.23
Moderate weight gain (5– < 10 kg)	19,495	1218	1.14	1.06–1.24	17,202	1 069	1.14	1.05–1.25
High weight gain (≥ 10 kg)	7486	464	1.19	1.06–1.32	6558	406	1.16	1.04–1.31
5 kg increment	80,930	4831	1.02	1.00–1.05	71,440	4 232	1.03	1.00–1.07
*Postmenopausal breast cancer* ^*b*^								
Weight loss (< −2kg)	5456	128	1.00	0.82–1.22	4040	97	1.16	0.92–1.47
Stable weight (−2– < 2 kg)	14,997	388	1.00	Reference	11,605	277	1.00	Reference
Low weight gain (2– < 5 kg)	12,462	383	1.11	0.97–1.28	9858	293	1.16	0.98–1.36
Moderate weight gain (5– < 10 kg)	10,103	312	1.08	0.93–1.25	8025	254	1.20	1.01–1.43
High weight gain (≥ 10 kg)	3690	121	1.15	0.93–1.41	2924	102	1.36	1.08–1.71
5 kg increment	46,708	1 332	1.04	0.98–1.09	36,452	1023	1.08	1.02–1.14
*Colorectal cancer* ^*c*^								
Weight loss (< −2kg)	7876	120	1.28	1.03–1.58	7874	120	1.25	1.01–1.55
Stable weight (−2– < 2 kg)	23,711	286	1.00	Reference	23,705	286	1.00	Reference
Low weight gain (2– < 5 kg)	22,362	273	1.11	0.94–1.31	22,361	273	1.11	0.94–1.32
Moderate weight gain (5– < 10 kg)	19,495	253	1.24	1.05–1.48	19,492	252	1.24	1.05–1.48
High weight gain (≥ 10 kg)	7486	75	1.04	0.8–1.34	7486	75	1.02	0.79–1.33
5 kg increment	80,930	1007	0.99	0.94–1.06	80,918	1006	1.00	0.94–1.06
*Colon cancer* ^*d*^								
Weight loss (< −2kg)	7876	91	1.30	1.01–1.66	7872	91	1.26	0.98–1.61
Stable weight (−2– < 2 kg)	23,711	212	1.00	Reference	23,695	210	1.00	Reference
Low weight gain (2– < 5 kg)	22,362	181	1.01	0.83–1.24	22,355	181	1.03	0.84–1.26
Moderate weight gain (5– < 10 kg)	19,495	174	1.19	0.97–1.47	19,487	173	1.19	0.97–1.46
High weight gain (≥ 10 kg)	7486	52	1.01	0.74–1.38	7483	52	0.98	0.72–1.34
5 kg increment	80,930	710	0.98	0.91–1.05	80,892	707	0.98	0.91–1.05
*Rectal cancer* ^*e*^								
Weight loss ( < −2kg)	7876	29	1.22	0.80–1.88	7876	29	1.22	0.80–1.88
Stable weight (−2 to < 2 kg)	23,711	74	1.00	Reference	23,711	74	1.00	Reference
Low weight gain (2 to < 5 kg)	22,362	92	1.37	1.00–1.86	22,362	92	1.37	1.00–1.86
Moderate weight gain (5 to < 10 kg)	19,495	79	1.38	1.00–1.91	19,495	79	1.38	1.00–1.91
High weight gain ( ≥ 10 kg)	7486	23	1.11	0.69–1.78	7486	23	1.11	0.69–1.78
5 kg increment	80,930	297	1.03	0.92–1.15	80,930	297	1.03	0.92–1.15
*Endometrial cancer* ^*f*^								
Weight loss (< −2kg)	7281	59	1.24	0.92–1.68	6813	55	1.03	0.75–1.41
Stable weight (−2– < 2 kg)	22,238	153	1.00	Reference	20,899	139	1.00	Reference
Low weight gain (2– < 5 kg)	20,998	136	0.94	0.75–1.19	19,798	127	0.99	0.78–1.26
Moderate weight gain (5– < 10 kg)	18,389	154	1.23	0.98–1.54	17,413	150	1.27	1.01–1.61
High weight gain (≥ 10 kg)	6989	69	1.51	1.13–2.01	6674	68	1.40	1.04–1.88
5 kg increment	75,895	571	1.10	1.02–1.19	71,597	539	1.12	1.04–1.20
*Ovarian cancer* ^*g*^								
Weight loss (< −2kg)	7614	37	1.62	1.09–2.41	6650	30	1.52	0.99–2.34
Stable weight (−2– < 2 kg)	23,041	74	1.00	Reference	20,409	66	1.00	Reference
Low weight gain (2– < 5 kg)	21,890	90	1.25	0.92–1.71	19,497	84	1.29	0.93–1.79
Moderate weight gain (5– < 10 kg)	19,133	84	1.32	0.96–1.81	16,955	75	1.30	0.93–1.82
High weight gain (≥ 10 kg)	7345	25	1.05	0.66–1.66	6511	23	1.08	0.67–1.74
5 kg increment	79,023	310	0.96	0.86–1.06	70,022	278	0.98	0.87–1.10
*Pancreatic cancer* ^*h*^								
Weight loss (< −2kg)	7876	25	1.84	1.12–3.02	7176	21	1.58	0.93–2.69
Stable weight (−2– < 2 kg)	23,711	42	1.00	Reference	21,697	39	1.00	Reference
Low weight gain (2– < 5 kg)	22,362	50	1.37	0.91–2.07	20,641	43	1.28	0.83–1.98
Moderate weight gain (5– < 10 kg)	19,495	48	1.59	1.04–2.43	18,124	46	1.60	1.03–2.47
High weight gain (≥ 10 kg)	7486	21	1.95	1.14–3.32	6971	21	1.91	1.11–3.30
5 kg increment	80,930	186	1.09	0.95–1.26	74,609	170	1.12	0.97–1.29
*Kidney cancer* ^*i*^								
Weight loss (< −2kg)	7876	17	1.29	0.73–2.28	5750	10	1.08	0.52–2.27
Stable weight (−2– < 2 kg)	23,711	41	1.00	Reference	18,350	26	1.00	Reference
Low weight gain (2– < 5 kg)	22,362	40	1.07	0.69–1.66	17,697	27	1.14	0.66–1.96
Moderate weight gain (5– < 10 kg)	19,495	35	1.10	0.70–1.74	15,223	23	1.14	0.64–2.01
High weight gain (≥ 10 kg)	7486	15	1.31	0.72–2.38	5634	8	1.10	0.49–2.45
5 kg increment	80,930	148	1.05	0.90–1.23	62,654	94	1.09	0.90–1.31

The Norwegian Women and Cancer study, 1991–2014 (*n* = 80,930)

^a^Model 2 for obesity-related cancer was adjusted for age, BMI (Q1), physical activity (Q1), smoking status and smoking transition

^b^Only in women who were postmenopausal at Q2, model 2 for postmenopausal breast cancer was adjusted for age, education, parity/age at first full-term pregnancy, hormone therapy use and history of breast cancer in the mother

^c^Model 2 for colorectal cancer was adjusted for age and smoking status

^d^Model 2 for colon cancer was adjusted for age, BMI (Q1) and smoking status

^e^Model 2 for rectal cancer did not significantly differ from model 1 and was only adjusted for age

^f^Model 2 for endometrial cancer was adjusted for age, BMI (Q1), age at menarche, parity/age at first full-term pregnancy, oral contraceptive use and menopausal status

^g^Model 2 for ovarian cancer was adjusted for age, physical activity (Q1) and parity/age at first full-term pregnancy

^h^Model 2 for pancreatic cancer was adjusted for age, education and smoking status

^i^Model 2 for kidney cancer was adjusted for age, alcohol intake and diabetes

**Fig. 3:**
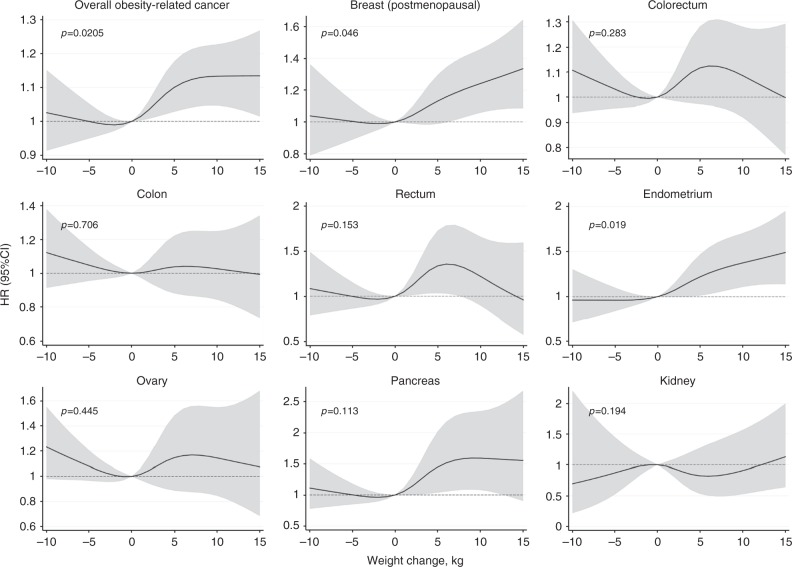
Non-linear effects of 6 years weight change and risk of specific and overall obesity-related cancers, with 95% CI. Restricted cubic splines were fitted with knots at −5, 1, 4 and 11 kg. *P* values are for non-linearity

When we allowed for non-linearity, we found a clear dose–response relationship with increasing weight gain for overall obesity-related cancer, postmenopausal breast cancer, endometrial and pancreatic cancer (Fig. 3). The increase in risk for these cancers was significant already with low or moderate weight gain (Supplementary Information, Table S6–9). There was no evidence of a significant interaction between BMI and weight change category in relation to overall and specific obesity-related cancer risk, which was further confirmed by the stratified analysis (Supplementary Information, Table S10). In addition, we found no significant interactions between HT use or menopausal status and weight change category.

## Discussion

In this study, we assessed the relationship between BMI, weight change and obesity-related cancer risk in a large and nationally representative cohort of women in Norway. We found that overweight and obesity increased overall obesity-related cancer risk by 9 and 24%. Furthermore, weight gain < 10 kg over 6 years, increased obesity-related cancer risk by 14%, whereas gaining 10 kg or more increased the risk by 16%, independent of BMI status at baseline. These findings highlight the health risks of excess body weight and increase in body weight among middle-aged women in Norway. Thus, maintaining stable weight is of utmost importance for the prevention of overall obesity-related cancer, especially as the increase in risk started at low levels of weight gain and most women gained weight. As in other studies, we found clear evidence of a significant association between excess body weight and postmenopausal breast, colorectal, colon, endometrial and kidney cancer,^2^ but no significant association with rectal, ovarian or pancreatic cancer. In addition, we found significant associations between weight gain and postmenopausal breast, colorectal, rectal, endometrial and pancreatic cancer but not between weight gain and ovarian and kidney cancer. These results suggest a similar effect of excess body weight and weight gain on hormone-related cancers (postmenopausal breast, endometrial and ovarian cancer), but a differential effect on kidney, colon, rectal and pancreatic cancer. Excess body weight and weight gain may affect organs differently, depending on the mechanism of cancer development.^19^ For instance, pancreatic cancer was not significantly associated with excess body weight, but there was a significant positive association with moderate and high weight gain. Pancreatic cancer development could be related to increased insulin levels and higher bioavailability of insulin-like growth factor,^20^ in which weight gain, rather than BMI, may play a more essential role. Our findings on weight gain and pancreatic cancer is novel. To the best of our knowledge, there has only been one previous study that included a separate analysis of pancreatic cancer and weight change in women, and it showed a non-significant, negative association.^21^ Another study including both women and men, demonstrated a non-significant, positive association.^22^ These two studies were included in a recent meta-analysis of weight gain and several cancers, wherein the authors hypothesised that in the presence of strong risk factors such as smoking, weight gain is not able to establish itself as an individual risk factor for pancreatic cancer.^4^ Our study sample included 170 pancreatic cancer cases, and we showed a significant association of moderate and high weight gain with pancreatic cancer risk, which remained after including smoking and smoking transition as potential confounders. Thus, our results suggest a possible role of weight development in the aetiology of pancreatic cancer, which must be confirmed by future studies, particularly among women. Kidney cancer is also an obesity-related cancer less-commonly diagnosed and we found only one previous study on weight change and kidney cancer in women.^23^ This aforementioned study showed no association with weight gain, consistent with our findings. On the contrary, obesity is reported as a strong predictor of kidney cancer,^2^ which is in line with our results of a 95% increased risk of kidney cancer among women with obesity.

Obesity, moderate and high weight gain were significantly associated with increased risk of postmenopausal breast cancer, which is in accordance with previous studies.^4,24^ The risk of postmenopausal breast cancer was higher in women experiencing moderate and high weight gain than among women with obesity, suggesting that weight gain may have an influence on postmenopausal breast cancer development beyond that of body composition. In our study, overweight, but not obesity, was associated with an increased risk of colorectal/colon cancer. This result may have been influenced by reverse causality, namely that weight loss was an early, pre-clinical symptom of colorectal cancer. There is inconsistency across studies on the association between weight change and colorectal cancer in women, with different results for colon and rectal cancers, but an overall indication of no association.^4,25^ We found a positive significant association between weight loss and moderate weight gain and colorectal cancer, but there was no significant association between high weight gain and colorectal cancer. For rectal cancer, we found a significant association for low and moderate weight gain but not high weight gain. Although we excluded all women with follow-up < 2 years, we can still not entirely rule out reverse causality, as we cannot differentiate between intentional and unintentional weight loss. In fact, studies of cancer incidence in women with obesity who have undergone bariatric surgery, show a decrease in overall and female-specific (breast and gynaecological) cancer risk compared with controls, suggesting that intentional weight loss may decrease cancer risk.^26^ However, large observational prospective cohort studies that can differentiate intentional and unintentional weight loss are warranted to improve our understanding of the effect of weight loss on cancer risk.

Endometrial cancer was strongly associated with obesity with a threefold elevated risk compared with women in normal weight. Moderate and high weight gain also increased the risk of endometrial cancer but the association for weight gain was not as strong as that for excess body weight. The evidence for a positive association between obesity, weight change and endometrial cancer risk is consistent with other studies.^24,27,28^ However, many studies on weight gain and endometrial cancer risk reported an increased risk only for substantially higher weight gain categories than those included in our study,^29–31^ whereas we report an increased risk starting at moderate weight gain.

The main strength of our study is its large, nationally representative, population-based sample of women in Norway with long follow-up time. The comprehensive questionnaires enabled us to control for important confounders such as anthropometric, sociodemographic, lifestyle, reproductive and menopausal factors, and the linkage with the Norwegian Cancer Registry provided us with virtually complete cancer case ascertainment. Thanks to the sample size and the extensiveness of the Norwegian Cancer Registry, we had the possibility to assess overall obesity-related cancer, and both common and less-common site-specific obesity-related cancers. There have been very few published articles on weight change and incidence of pancreatic and kidney cancer in women, and here we have added evidence to the current literature. Nevertheless, this study has several limitations. Height and weight were self-reported, and there is a well-established tendency to overestimate height as well as underestimate weight that increases with age and BMI.^32^ In our study, we assume that the potential misclassification due to this information bias was non-differential between cases and non-cases. Therefore, our risk estimates may have been underestimated. Furthermore, a validation study of BMI has been conducted in the NOWAC study and showed substantial agreement between self-reports and objective measurements.^33^ In addition, the covariate physical activity was also self-reported and displayed a moderate significant correlation with heart rate and movement in a previous validation study.^34^ Total energy intake was omitted from the analyses because the food-frequency questionnaire was not provided to all participants in this study, leading to a large amount of missing data (61%), and because of known biases with respect to obesity.^35^ Finally, as mentioned above, the lack of information on intentionality of weight loss to avoid reverse causality hampered the weight loss analysis.

The mean BMI in our study sample was 24.1. Thus, our study sample is slimmer than in many other high-income countries.^36^ The generalisability of our study is restricted to women in Norway but it is unlikely that the association between excess body weight/weight gain and obesity-related cancer substantially differs across regions. However, the impact of our findings, i.e., the number of cancer cases attributable to excess body weight and weight gain (given a causal relationship) may potentially be larger in regions with higher prevalence of excess body weight or higher weight gain.

In summary, maintaining stable weight in middle adulthood, regardless BMI status, and avoiding excess body weight are important for the prevention of several obesity-related cancers. We found strong associations between obesity and endometrial cancer risk, and high weight gain and pancreatic cancer risk. Our findings on weight gain and pancreatic cancer risk are particularly interesting given the increasing incidence of pancreatic cancer in women in Norway, and the very poor prognosis of the disease.^8^ If our findings are confirmed, avoidance of weight gain could be considered a potential preventive measure for pancreatic cancer.

## Electronic supplementary material

Supplemental material 1

Supplemental material 2

Supplemental material 3

Supplemental material 4

## Availability of data and material

For the data supporting the presented results, please contact the person responsible for the NOWAC study—https://site.uit.no/nowac/contact-information/.
