# Supplementary material for: Excess body weight, weight gain and obesity-related cancer risk in women in Norway: the Norwegian Women and Cancer study
Source: Br J Cancer. 2018 Sep 11;119(5):646–56. doi: 10.1038/s41416-018-0240-5 (PMC6162329; doi:10.1038/s41416-018-0240-5)
Supplement: Supplementary file 1 — Supplemental material 1 [file 41416_2018_240_MOESM1_ESM.docx]

Supplementary Information

Supplementary information Table 1 presents population characteristics by weight change category in PDF file format.

**Table 1.** Population characteristics by weight change category between enrolment questionnaire (Q1) and the follow-up questionnaire (Q2). The Norwegian Women and Cancer study, 1991-2011 (n=80 930)

|  | Weight change category (kg) | | | | | |
| --- | --- | --- | --- | --- | --- | --- |
|  | N† | Weight loss  (<-2kg) | Stable weight  (-2 to <2kg) | Low weight gain  (2 to <5kg) | Moderate weight gain  (5 to <10kg) | High weight gain  (≥10kg) |
| Number of women. n (%) | 80 930 | 7 876 (9.7) | 23 711 (29.3) | 22 362 (27.6) | 19 495 (24.1) | 7 486 (9.3) |
| Obesity-related cancer. n | 80 930 | 478 | 1 315 | 1 356 | 1 218 | 464 |
| Characteristics* |  |  |  |  |  |  |
| Age (y). mean (SD) | 80 930 | 55.3 (9.3) | 53.9 (9.0) | 52.0 (8.1) | 50.8 (7.5) | 49.8 (7.0) |
| Body mass index (kg/m). mean (SD) (Q1) | 80 904 | 26.2 (4.8) | 23.5 (3.5) | 23.0 (3.1) | 23.6 (3.4) | 24.5 (4.0) |
| Education (y). % | 77 415 |  |  |  |  |  |
| <10 |  | 31.1 | 25.4 | 22.6 | 23.3 | 25.7 |
| 10-12 |  | 23.7 | 22.9 | 23.3 | 25.0 | 25.3 |
| >12 |  | 45.1 | 51.7 | 54.1 | 51.7 | 49.0 |
| Physical activity level. % (Q1) | 74 097 |  |  |  |  |  |
| Low |  | 32.8 | 23.8 | 22.9 | 26.8 | 31.7 |
| Moderate |  | 39.5 | 42.6 | 43.0 | 42.5 | 38.9 |
| High |  | 27.7 | 33.6 | 34.0 | 30.7 | 29.4 |
| Smoking status. % | 80 918 |  |  |  |  |  |
| Never smoker |  | 33.5 | 39.1 | 39.9 | 37.6 | 31.9 |
| Former smoker |  | 31.4 | 32.4 | 34.9 | 36.9 | 42.3 |
| Current smoker |  | 35.1 | 28.5 | 25.3 | 25.6 | 25.9 |
| Alcohol intake (g/day). median | 79 349 | 1.4 | 1.6 | 1.9 | 1.8 | 1.5 |
| Age at menarche (y). mean (SD) (Q1) | 79 788 | 13.2 (1.4) | 13.4 (1.4) | 13.3 (1.4) | 13.3 (1.4) | 13.1 (1.4) |
| Age at first full-term pregnancy (y). mean (SD) | 74 062 | 23.7 (4.5) | 24.2 (4.4) | 24.1 (4.3) | 24.0 (4.4) | 23.7 (4.5) |
| Parity. % | 80 930 |  |  |  |  |  |
| Nulliparous |  | 8.3 | 8.3 | 7.9 | 7.6 | 9.1 |
| 1-2 children |  | 49.0 | 51.3 | 53.2 | 54.6 | 53.0 |
| ≥ 3 children |  | 42.8 | 40.4 | 38.9 | 37.8 | 37.8 |
| Oral contraceptive use. % | 80 004 |  |  |  |  |  |
| Never |  | 48.7 | 45.7 | 42.4 | 39.4 | 38.1 |
| Ever |  | 51.3 | 54.3 | 57.6 | 60.6 | 61.9 |
| Menopausal status. % | 80 930 |  |  |  |  |  |
| Premenopausal |  | 44.2 | 53.1 | 62.5 | 67.8 | 70.3 |
| Perimenopausal |  | 5.0 | 4.3 | 4.0 | 4.0 | 3.9 |
| Postmenopausal |  | 43.5 | 36.0 | 26.5 | 21.4 | 18.3 |
| Unknown |  | 7.3 | 6.7 | 6.9 | 6.8 | 7.6 |
| Age at menopause (y). mean (SD) | 45 881 | 48.7 (5) | 49.0 (4.7) | 48.9 (4.8) | 48.6 (4.9) | 47.9 (5.2) |
| Hormone therapy use. % | 80 930 |  |  |  |  |  |
| Never |  | 61.1 | 63.4 | 65.4 | 66.0 | 66.1 |
| Former |  | 18.2 | 14.1 | 12.3 | 12.0 | 13.2 |
| Current |  | 20.7 | 22.5 | 22.3 | 22.0 | 20.7 |
|  |  |  |  |  |  |  |
| Characteristics transition Q1 🡪 Q2 |  |  |  |  |  |  |
| Physical activity level. % | 67 737 |  |  |  |  |  |
| Increase |  | 29.6 | 24.3 | 21.9 | 20.6 | 16.6 |
| Decrease |  | 20.1 | 22.9 | 26.0 | 28.7 | 35.6 |
| No change |  | 50.3 | 52.9 | 52.0 | 50.7 | 47.8 |
| Smoking status. % | 78 008 |  |  |  |  |  |
| Cessation |  | 5.4 | 5.7 | 7.8 | 11.8 | 19.3 |
| Restart |  | 7.0 | 4.6 | 4.2 | 4.1 | 4.1 |
| No change |  | 87.6 | 89.7 | 88.0 | 84.1 | 76.6 |
| Menopausal status. % | 80 930 |  |  |  |  |  |
| No transition to menopause |  | 51.7 | 43.2 | 34.1 | 29.0 | 26.8 |
| Transition to menopause |  | 48.3 | 56.8 | 65.9 | 71.0 | 73.2 |

*Overall differences between weight change categories were significant for all variables (p<0.001)

†N is the total amount of responses for the specific variable

Abbreviations: y: years, SD: standard deviation
