# Supplementary material for: Excess body weight, weight gain and obesity-related cancer risk in women in Norway: the Norwegian Women and Cancer study
Source: Br J Cancer. 2018 Sep 11;119(5):646–56. doi: 10.1038/s41416-018-0240-5 (PMC6162329; doi:10.1038/s41416-018-0240-5)
Supplement: Supplementary file 2 — Supplemental material 2 [file 41416_2018_240_MOESM2_ESM.docx]

Supplementary Information

Supplementary information Table 2 presents stratified analysis of endometrial cancer risk and body mass index by menopausal status in PDF file format.

**Table 2.** Hazard ratio (HR) with 95% confidence interval (CI) for endometrial cancer risk by body mass index (BMI) and stratified by menopausal status. The Norwegian Women and Cancer study, 1991-2014 (n=113 150)*

|  | Menopausal status | | | | | | | | | | | | | | | |
| --- | --- | --- | --- | --- | --- | --- | --- | --- | --- | --- | --- | --- | --- | --- | --- | --- |
|  | Premenopausal | | | | Perimenopausal | | | | Postmenopausal | | | | Unknown | | | |
|  | N | Cancer cases | HR | 95% CI | N | Cancer cases | HR | 95% CI | N | Cancer cases | HR | 95% CI | N | Cancer cases | HR | 95% CI |
| BMI category |  |  |  |  |  |  |  |  |  |  |  |  |  |  |  |  |
| Underweight | 1 766 | 7 | 0.65 | 0.30-1.37 | 115 | 0 | 0.00 | 0.00-0.00 | 619 | 3 | 0.80 | 0.25-2.50 | 2 | 0 | 0.00 | 0.00-0.00 |
| Normal weight | 44 204 | 297 | 1.00 | Reference | 3 663 | 25 | 1.00 | Reference | 22 663 | 146 | 1.00 | Reference | 3 709 | 21 | 1.00 | Reference |
| Overweight | 11 658 | 131 | 1.58 | 1.28-1.94 | 1 708 | 13 | 1.11 | 0.56-2.18 | 13 252 | 130 | 1.49 | 1.17-1.89 | 1 373 | 3 | 0.37 | 0.11-1.25 |
| Obesity | 2 994 | 46 | 2.12 | 1.54-2.91 | 595 | 20 | 4.74 | 2.56-8.78 | 4 330 | 85 | 3.12 | 2.37-4.10 | 407 | 5 | 2.25 | 0.82-6.15 |
| 5 BMI increment | 60 622 | 481 | 1.42 | 1.28-1.58 | 6 081 | 58 | 1.70 | 1.42-2.03 | 40 864 | 364 | 1.55 | 1.43-1.69 | 5 583 | 29 | 1.29 | 0.88-1.88 |

* Adjusted for age, education, age at menarche, parity/age at first full-term pregnancy, and oral contraceptive use
