# Supplementary material for: Excess body weight, weight gain and obesity-related cancer risk in women in Norway: the Norwegian Women and Cancer study
Source: Br J Cancer. 2018 Sep 11;119(5):646–56. doi: 10.1038/s41416-018-0240-5 (PMC6162329; doi:10.1038/s41416-018-0240-5)
Supplement: Supplementary file 3 — Supplemental material 3 [file 41416_2018_240_MOESM3_ESM.docx]

Supplementary Information

Supplementary information Table 3-9 presents tables of specific and overall obesity-related cancers with a clear dose-response relationship and their respective hazard ratios and confidence intervals for selected values. The tables complement Figure 2 (body mass index analysis) and Figure 3 (weight change analysis) in the original research article and is presented in PDF file format.

# Body mass index analysis

**Table 3.** Hazard ratio (HR) with 95% confidence interval (CI) for overall obesity-related cancer risk by body mass index (BMI) values, with fitted restricted cubic splines at knots BMI 19, 22, 25, and 31

| Selected  BMI values, kg/m^2^ | HR | 95%CI |
| --- | --- | --- |
| 15 | 1.00 | Reference |
| 16 | 1.00 | 0.99-1.06 |
| 17 | 1.03 | 0.99-1.13 |
| 18 | 1.06 | 0.98-1.20 |
| 19 | 1.08 | 0.98-1.27 |
| 20 | 1.11 | 0.97-1.35 |
| 21 | 1.14 | 0.97-1.42 |
| 22 | 1.17 | 0.98-1.48 |
| 23 | 1.20 | 0.99-1.51 |
| 24 | 1.22 | 1.01-1.52 |
| 25 | 1.24 | 1.04-1.53 |
| 26 | 1.26 | 1.06-1.55 |
| 27 | 1.28 | 1.08-1.57 |
| 28 | 1.30 | 1.10-1.60 |
| 29 | 1.33 | 1.12-1.63 |
| 30 | 1.35 | 1.14-1.67 |
| 31 | 1.38 | 1.16-1.70 |
| 32 | 1.41 | 1.18-1.74 |
| 33 | 1.43 | 1.20-1.78 |
| 34 | 1.46 | 1.21-1.83 |
| 35 | 1.49 | 1.23-1.87 |
| 36 | 1.52 | 1.25-1.92 |
| 37 | 1.55 | 1.26-1.97 |
| 38 | 1.58 | 1.28-2.02 |
| 39 | 1.61 | 1.30-2.08 |
| 40 | 1.64 | 1.31-2.13 |
| 41 | 1.67 | 1.33-2.19 |
| 42 | 1.71 | 1.34-2.25 |
| 43 | 1.74 | 1.36-2.32 |
| 44 | 1.77 | 1.37-2.38 |
| 45 | 1.81 | 1.39-2.45 |

**Table 4.** Hazard ratio (HR) with 95% confidence interval (CI) for endometrial cancer risk by selected body mass index (BMI) values, with fitted restricted cubic splines at knots BMI 19, 22, 25, and 31, for selected values

| Selected  BMI values,  kg/m^2^ | HR | 95%CI |
| --- | --- | --- |
| 15 | 1.00 | Reference |
| 16 | 1.08 | 0.97-1.21 |
| 17 | 1.17 | 0.94-1.46 |
| 18 | 1.26 | 0.91-1.76 |
| 19 | 1.37 | 0.88-2.13 |
| 20 | 1.48 | 0.85-2.56 |
| 21 | 1.60 | 0.84-3.04 |
| 22 | 1.75 | 0.87-3.51 |
| 23 | 1.92 | 0.94-3.90 |
| 24 | 2.11 | 1.05-4.22 |
| 25 | 2.32 | 1.18-4.54 |
| 26 | 2.54 | 1.32-4.88 |
| 27 | 2.77 | 1.45-5.27 |
| 28 | 3.01 | 1.59-5.70 |
| 29 | 3.26 | 1.72-6.17 |
| 30 | 3.53 | 1.86-6.68 |
| 31 | 3.81 | 2.01-7.25 |
| 32 | 4.12 | 2.16-7.86 |
| 33 | 4.46 | 2.33-8.54 |
| 34 | 4.82 | 2.51-9.27 |
| 35 | 5.21 | 2.70-10.07 |
| 36 | 5.63 | 2.90-10.95 |
| 37 | 6.09 | 3.12-11.91 |
| 38 | 6.59 | 3.35-12.96 |
| 39 | 7.12 | 3.59-14.11 |
| 40 | 7.70 | 3.86-15.37 |
| 41 | 8.32 | 4.14-16.75 |
| 42 | 9.00 | 4.44-18.26 |
| 43 | 9.73 | 4.75-19.91 |
| 44 | 10.52 | 5.09-21.72 |
| 45 | 11.37 | 5.46-23.71 |

**Table 5.** Hazard ratio (HR) with 95% confidence interval (CI) for kidney cancer risk by selected body mass index (BMI) values, with fitted restricted cubic splines at knots BMI 19, 22, 25, and 31

| Selected  BMI values,  kg/m^2^ | HR | 95%CI |
| --- | --- | --- |
| 15 | 1.00 | Reference |
| 16 | 1.16 | 0.92-1.46 |
| 17 | 1.34 | 0.84-2.14 |
| 18 | 1.55 | 0.77-3.12 |
| 19 | 1.80 | 0.71-4.56 |
| 20 | 2.08 | 0.65-6.62 |
| 21 | 2.37 | 0.62-9.14 |
| 22 | 2.64 | 0.61-11.45 |
| 23 | 2.84 | 0.64-12.64 |
| 24 | 2.99 | 0.69-12.86 |
| 25 | 3.11 | 0.76-12.74 |
| 26 | 3.24 | 0.82-12.83 |
| 27 | 3.39 | 0.88-13.16 |
| 28 | 3.56 | 0.93-13.68 |
| 29 | 4.00 | 0.98-14.38 |
| 30 | 4.00 | 1.03-15.22 |
| 31 | 4.00 | 1.07-16.20 |
| 32 | 4.00 | 1.12-17.28 |
| 33 | 5.00 | 1.17-18.46 |
| 34 | 5.00 | 1.21-19.75 |
| 35 | 5.17 | 1.26-21.17 |
| 36 | 5.45 | 1.31-22.73 |
| 37 | 5.75 | 1.35-24.43 |
| 38 | 6.06 | 1.40-26.30 |
| 39 | 6.40 | 1.44-28.35 |
| 40 | 6.75 | 1.49-30.60 |
| 41 | 7.12 | 1.53-33.07 |
| 42 | 8.00 | 1.58-35.78 |
| 43 | 8.00 | 1.62-38.76 |
| 44 | 8.00 | 1.66-42.03 |
| 45 | 9.00 | 1.70-45.63 |

# Weight change analysis

**Table 6.** Hazard ratio (HR) with 95% confidence interval (CI) for overall obesity-related cancer risk by selected weight change values, with fitted restricted cubic splines at knots -5, 1, 4, and 11 kg

| Selected  weight change  values, kg | HR | 95%CI |
| --- | --- | --- |
| -10 | 1.03 | 0.91-1.15 |
| -9 | 1.02 | 0.92-1.13 |
| -8 | 1.02 | 0.93-1.11 |
| -7 | 1.01 | 0.94-1.09 |
| -6 | 1.00 | 0.94-1.07 |
| -5 | 1.00 | 0.95-1.05 |
| -4 | 0.99 | 0.96-1.03 |
| -3 | 0.99 | 0.96-1.02 |
| -2 | 0.99 | 0.97-1.01 |
| -1 | 0.99 | 0.98-1.00 |
| 0 | 1.00 | Reference |
| 1 | 1.01 | 1.00-1.03 |
| 2 | 1.03 | 1.01-1.06 |
| 3 | 1.06 | 1.01-1.10 |
| 4 | 1.08 | 1.02-1.15 |
| 5 | 1.10 | 1.03-1.18 |
| 6 | 1.11 | 1.04-1.20 |
| 7 | 1.12 | 1.04-1.21 |
| 8 | 1.13 | 1.05-1.22 |
| 9 | 1.13 | 1.05-1.22 |
| 10 | 1.13 | 1.05-1.23 |
| 11 | 1.13 | 1.04-1.23 |
| 12 | 1.13 | 1.04-1.24 |
| 13 | 1.13 | 1.03-1.25 |
| 14 | 1.13 | 1.02-1.26 |
| 15 | 1.13 | 1.01-1.27 |

**Table 7.** Hazard ratio (HR) with 95% confidence interval (CI) for postmenopausal breast cancer risk by selected weight change values, with fitted restricted cubic splines at knots -5, 1, 4, and 11 kg

| Selected  weight change  values, kg | HR | 95%CI |
| --- | --- | --- |
| -10 | 1.04 | 0.79-1.36 |
| -9 | 1.03 | 0.81-1.31 |
| -8 | 1.02 | 0.83-1.26 |
| -7 | 1.02 | 0.85-1.22 |
| -6 | 1.01 | 0.87-1.17 |
| -5 | 1.00 | 0.89-1.13 |
| -4 | 1.00 | 0.91-1.09 |
| -3 | 0.99 | 0.93-1.06 |
| -2 | 0.99 | 0.95-1.03 |
| -1 | 0.99 | 0.97-1.01 |
| 0 | 1.00 | Reference |
| 1 | 1.02 | 0.99-1.04 |
| 2 | 1.04 | 0.99-1.10 |
| 3 | 1.07 | 0.98-1.17 |
| 4 | 1.10 | 0.98-1.24 |
| 5 | 1.13 | 0.99-1.30 |
| 6 | 1.16 | 1.00-1.34 |
| 7 | 1.18 | 1.01-1.38 |
| 8 | 1.20 | 1.03-1.41 |
| 9 | 1.22 | 1.04-1.43 |
| 10 | 1.24 | 1.06-1.46 |
| 11 | 1.26 | 1.07-1.48 |
| 12 | 1.28 | 1.08-1.52 |
| 13 | 1.30 | 1.08-1.55 |
| 14 | 1.32 | 1.08-1.60 |
| 15 | 1.33 | 1.08-1.64 |

**Table 8.** Hazard ratio (HR) with 95% confidence interval (CI) for endometrial cancer risk by selected weight change values, with fitted restricted cubic splines at knots -5, 1, 4, and 11 kg

| Selected  weight change  values, kg | HR | 95%CI |
| --- | --- | --- |
| -10 | 0.96 | 0.71-1.30 |
| -9 | 0.96 | 0.74-1.26 |
| -8 | 0.96 | 0.76-1.22 |
| -7 | 0.96 | 0.79-1.18 |
| -6 | 0.96 | 0.81-1.14 |
| -5 | 0.96 | 0.84-1.10 |
| -4 | 0.96 | 0.87-1.06 |
| -3 | 0.96 | 0.90-1.04 |
| -2 | 0.97 | 0.92-1.01 |
| -1 | 0.98 | 0.96-1.00 |
| 0 | 1.00 | Reference |
| 1 | 1.03 | 1.00-1.06 |
| 2 | 1.07 | 1.00-1.15 |
| 3 | 1.12 | 1.00-1.26 |
| 4 | 1.17 | 1.00-1.37 |
| 5 | 1.22 | 1.01-1.47 |
| 6 | 1.26 | 1.03-1.54 |
| 7 | 1.29 | 1.05-1.60 |
| 8 | 1.32 | 1.07-1.64 |
| 9 | 1.35 | 1.09-1.67 |
| 10 | 1.37 | 1.11-1.70 |
| 11 | 1.40 | 1.12-1.74 |
| 12 | 1.42 | 1.13-1.78 |
| 13 | 1.44 | 1.14-1.83 |
| 14 | 1.47 | 1.14-1.88 |
| 15 | 1.49 | 1.14-1.95 |

**Table 9.** Hazard ratio (HR) with 95% confidence interval (CI) for pancreatic cancer risk by selected weight change values, with fitted restricted cubic splines at knots -5, 1, 4, and 11 kg

| Selected  weight change  values, kg | HR | 95%CI |
| --- | --- | --- |
| -10 | 1.11 | 0.78-1.59 |
| -9 | 1.09 | 0.79-1.49 |
| -8 | 1.07 | 0.81-1.41 |
| -7 | 1.04 | 0.82-1.33 |
| -6 | 1.02 | 0.84-1.25 |
| -5 | 1.00 | 0.85-1.18 |
| -4 | 0.98 | 0.86-1.12 |
| -3 | 0.97 | 0.88-1.07 |
| -2 | 0.96 | 0.90-1.03 |
| -1 | 0.97 | 0.93-1.01 |
| 0 | 1.00 | Reference |
| 1 | 1.05 | 1.00-1.11 |
| 2 | 1.14 | 1.00-1.30 |
| 3 | 1.25 | 1.01-1.54 |
| 4 | 1.36 | 1.02-1.80 |
| 5 | 1.45 | 1.04-2.02 |
| 6 | 1.52 | 1.06-2.17 |
| 7 | 1.56 | 1.07-2.27 |
| 8 | 1.58 | 1.08-2.31 |
| 9 | 1.59 | 1.09-2.33 |
| 10 | 1.59 | 1.08-2.35 |
| 11 | 1.58 | 1.06-2.37 |
| 12 | 1.58 | 1.03-2.42 |
| 13 | 1.57 | 0.99-2.48 |
| 14 | 1.56 | 0.95-2.57 |
| 15 | 1.55 | 0.90-2.67 |
