# Supplementary material for: Excess body weight, weight gain and obesity-related cancer risk in women in Norway: the Norwegian Women and Cancer study
Source: Br J Cancer. 2018 Sep 11;119(5):646–56. doi: 10.1038/s41416-018-0240-5 (PMC6162329; doi:10.1038/s41416-018-0240-5)
Supplement: Supplementary file 4 — Supplemental material 4 [file 41416_2018_240_MOESM4_ESM.docx]

Supplementary Information

Supplementary information Table 10 presents stratified analysis of overall obesity-related cancer risk and weight change by body mass index status in PDF file format.

**Table 10.** Hazard ratio (HR) with 95% confidence interval (CI) for obesity-related cancer risk by weight change category between the enrolment (Q1) and follow-up questionnaire (Q2) stratified by body mass index (BMI) status (Q1). The Norwegian Women and Cancer study, 1991-2014 (n=71 440)*

|  | BMI category | | | | | | | | | | | | | | | |
| --- | --- | --- | --- | --- | --- | --- | --- | --- | --- | --- | --- | --- | --- | --- | --- | --- |
|  | Underweight | | | | Normal weight | | | | Overweight | | | | Obesity | | | |
|  | N | Cancer cases | HR | 95% CI | N | Cancer cases | HR | 95% CI | N | Cancer cases | HR | 95% CI | N | Cancer cases | HR | 95% CI |
| Weight change category |  |  |  |  |  |  |  |  |  |  |  |  |  |  |  |  |
| Weight loss (<-2kg) | 58 | 5 | 1.13 | 0.43-2.98 | 3 191 | 161 | 1.04 | 0.88-1.24 | 2 442 | 152 | 1.00 | 0.82-1.22 | 1 195 | 88 | 1.36 | 0.99-1.89 |
| Stable weight (-2 to <2kg) | 557 | 30 | 1.00 | Reference | 14 863 | 754 | 1.00 | Reference | 4 499 | 296 | 1.00 | Reference | 1 031 | 62 | 1.00 | Reference |
| Low weight gain (2 to <5kg) | 609 | 22 | 0.75 | 0.43-1.32 | 15 091 | 915 | 1.19 | 1.08-1.31 | 3 520 | 228 | 1.01 | 0.85-1.20 | 624 | 44 | 1.15 | 0.78-1.70 |
| Moderate weight gain (5 to <10kg) | 388 | 22 | 1.21 | 0.68-2.15 | 12 163 | 743 | 1.19 | 1.07-1.32 | 3 817 | 238 | 0.98 | 0.82-1.17 | 834 | 66 | 1.39 | 0.98-1.96 |
| High weight gain (≥10kg) | 166 | 8 | 0.80 | 0.36-1.81 | 3 940 | 235 | 1.19 | 1.03-1.39 | 1 866 | 118 | 1.08 | 0.87-1.35 | 586 | 45 | 1.40 | 0.95-2.07 |
| 5 kg increment | 1 778 | 87 | 1.19 | 0.75-1.19 | 49 248 | 2 808 | 1.11 | 1.02-1.11 | 16 144 | 1 032 | 1.07 | 0.95-1.07 | 4 270 | 305 | 1.08 | 0.95-1.08 |

*Adjusted for age, physical activity (Q1), smoking status, and smoking transition­­­­
